# Persistently low serum alkaline phosphatase in adults: prevalence and clinical characteristics in a large tertiary care hospital

**DOI:** 10.1186/s13023-026-04493-1

**Published:** 2026-07-18

**Authors:** Fahad Alshahrani, Sara Alsuliman, Reema Alkalefah, Shaden Almutairi, Yara Aleidi, Mohammed Almohaya

**Affiliations:** 1 King Saud bin Abdulaziz University for Health Sciences, College of Medicine, Riyadh, Saudi Arabia; 2 King Abdullah International Medical Research Center (KAIMRC), Riyadh, Saudi Arabia; 3 King Abdulaziz Medical City, Ministry of National Guard Health Affairs, Riyadh, Saudi Arabia; 4 King Fahad Medical City, Riyadh, Saudi Arabia

**Keywords:** Alkaline phosphatase, Hypophosphatasia, Metabolic bone disease, Musculoskeletal pain, Biochemical marker

## Abstract

**Background:**

Alkaline phosphatase (ALP) plays an essential role in skeletal mineralization and bone metabolism. While elevated ALP levels are commonly investigated in clinical practice, persistently low ALP values are often overlooked despite their potential association with hypophosphatasia (HPP), a rare metabolic bone disorder caused by pathogenic variants in the *ALPL* gene. This study aimed to determine the prevalence and clinical characteristics of adults with persistently low ALP in a large tertiary care hospital.

**Methods:**

We conducted a retrospective review of adult patients who underwent serum ALP testing at King Abdulaziz Medical City, Riyadh, between January 2017 and December 2024. Patients with ≥ 2 ALP measurements < 40 IU/L separated by at least 30 days were identified. Secondary causes of low ALP were excluded through review of clinical history, laboratory data, and medication records. Demographic, clinical, biochemical, and imaging data were analyzed.

**Results:**

Among 243,362 adults with 928,403 ALP measurements, 7,307 patients (3.0%) had at least one ALP value < 40 IU/L. A total of 179 patients had ≥ 2 ALP measurements < 40 IU/L. After excluding 154 patients with secondary causes or incomplete data, 25 patients were identified with unexplained persistently low ALP (0.01% of the total tested population). The mean age was 52.2 years (range 18–92), and 68% were female. The mean nadir ALP was 30.2 IU/L. Musculoskeletal (MSK) symptoms (92%) and fatigue (79%) were the most common clinical manifestations, followed by dental abnormalities (50%), anxiety (33%), and depression (29%). Fractures were reported in 46% of patients, most commonly affecting the vertebrae, hips, and metatarsals. In subgroup analysis, patients with MSK symptoms had significantly lower nadir ALP levels (*p* = 0.021). Familial clustering of persistently low ALP was observed in 25% of patients. Notably, none of the reviewed electronic medical records documented recognition of persistently low ALP or consideration of hypophosphatasia.

**Conclusion:**

Persistently low ALP is uncommon but clinically relevant. Many affected patients demonstrated clinical features compatible with possible adult hypophosphatasia, and familial clustering suggests a possible genetic contribution. Greater awareness of this biochemical finding may improve recognition of potential HPP, prevent inappropriate antiresorptive therapy, and facilitate earlier diagnostic evaluation, including biochemical and genetic testing.

## Introduction

Alkaline phosphatase (ALP) is a critical enzyme involved in skeletal and mineral metabolism, with the bone and liver isoforms accounting for the majority of circulating activity in adults. In clinical practice, elevated ALP levels are routinely investigated, whereas persistently low values are often overlooked despite their potential association with hypophosphatasia (HPP), a rare genetic disorder caused by pathogenic variants in the *ALPL* gene encoding tissue-nonspecific alkaline phosphatase (TNSALP) [1].

The diagnosis of HPP relies on a combination of clinical features, biochemical abnormalities, and radiologic findings. Although genetic testing may support the diagnosis, not all patients with clinically suspected HPP have identifiable *ALPL* variants [2]. In adults, HPP often presents with nonspecific manifestations such as fatigue, recurrent fractures, musculoskeletal (MSK) pain, chondrocalcinosis, dental abnormalities, and impaired bone mineralization [3, 4]. Because these features overlap with more common metabolic bone and rheumatologic disorders, affected individuals are frequently misdiagnosed.

Screening for persistently low ALP in osteoporosis or metabolic bone clinics may help identify patients with possible HPP and prevent inappropriate antiresorptive therapy, which may increase the risk of complications such as atypical femoral fractures (AFF) [5]. In this context, persistently low ALP levels represent a critical diagnostic clue.

Epidemiological studies have reported prevalence rates of persistently low serum ALP in adults ranging from 0.06% to over 1%, substantially higher than the prevalence of genetically or clinically confirmed HPP [6–9]. For instance, a German population-based study of 6.9 million laboratory measurements found that 8.46% of samples showed ALP < 30 U/L and an additional 9.47% ranged between 30 and 40 U/L, suggesting that nearly one in five adult samples may fall below standard reference ranges [6]. In contrast, a rural American cohort of 885,165 adults demonstrated a prevalence of 0.06% for persistently low ALP [7], while a French tertiary hospital study (*n* = 48,755) reported a prevalence of 0.13%, with recognition rates as low as 3% [8]. Similarly, in a large Brazilian hospital cohort of 289,247 adults, 0.36% had ALP values < 40 U/L, and 0.14% had at least two confirmed low ALP < 40 U/L [9].

Severe forms of HPP are exceedingly rare, with prevalence estimates ranging from 1 in 100,000 to 1 in 300,000, whereas moderate forms may affect as many as 1 in 6,370 individuals and up to 1 in 500 for the mild adult form [4]. The notable discrepancy between the frequency of persistently low ALP and the rarity of confirmed HPP underscores the likelihood of substantial underdiagnosis. This highlights the need for improved clinical recognition, systematic evaluation, and better characterization of individuals with persistently reduced ALP activity.

Accordingly, this study aimed to determine the prevalence and clinical recognition of persistently low ALP among adults in a large tertiary care hospital. In addition, we sought to characterize the clinical, biochemical, and imaging features of these patients to better understand the potential diagnostic gap in identifying possible HPP. To our knowledge, this is the first study to systematically evaluate the prevalence and clinical characteristics of persistently low ALP in an adult Arab population from the Middle East, a demographic underrepresented in the global HPP literature. Additionally, this study provides real-world evidence of a critical diagnostic gap in a large tertiary care setting and documents familial clustering of low ALP in a non-European population, supporting a potential genetic contribution and underscoring the importance of systematic family screening.

## Methods

This study was conducted at King Abdulaziz Medical City, Riyadh (KAMC-R), a 1,500-bed tertiary care hospital in Saudi Arabia that serves National Guard employees and their dependents. The facility provides comprehensive services across all medical subspecialties.

We performed a retrospective chart review between January 1, 2017, and December 31, 2024. During this period, a total of 243,362 adult patients had at least one serum ALP assessment. Among these, 7,307 patients had at least one ALP measurement below 40 IU/L, which corresponds to the lower limit of the adult reference range at KAMC-R. Serum ALP measurements were performed using an enzymatic colorimetric assay on the Abbott Architect platform. The ALP reference intervals used in this study were those established at KAMC-R on the Abbott Architect platform: for adults aged 25 years or older, the reference range is 40–150 IU/L (both sexes); for individuals aged 18–24 years, age-specific thresholds of 59–165 IU/L (males) and 48–95 IU/L (females) apply. Accordingly, persistently low ALP was defined as two or more measurements below these lower reference limits, separated by at least 30 days.

Of these patients, 179 had two or more ALP measurements below 40 IU/L and complete clinical data and/or available dual-energy X-ray absorptiometry (DXA) results. Medical records for these patients were reviewed to identify potential secondary causes of low ALP levels other than HPP, including hypothyroidism, malnutrition, celiac disease, sepsis, recent surgery, hemorrhage, anemia, hypomagnesemia, zinc deficiency, lung and/or cardiac transplantation, plasma exchange, and the use of medications such as corticosteroids, chemotherapy, and antiresorptive agents. Secondary causes were identified based on clinical documentation, laboratory parameters, and medication history recorded in the electronic medical record.

For each patient, all recorded ALP values were reviewed, including the lowest, highest, and mean levels, to avoid misinterpretation that could arise from relying solely on the lowest value. Persistently low ALP was defined as ≥ 2 measurements < 40 IU/L separated by at least 30 days [7, 9]. Patients meeting this definition were selected for further evaluation. The inclusion, exclusion, and elimination criteria applied at each stage were as follows. Inclusion criteria: adult patients (≥ 18 years) with ≥ 2 serum ALP measurements below the lower limit of the reference range (< 40 IU/L for patients ≥ 25 years; age-specific thresholds applied for younger adults) separated by at least 30 days, and with sufficient clinical documentation for review. Exclusion criteria: patients with identifiable secondary causes of low ALP, including hypothyroidism, malnutrition, celiac disease, sepsis, recent surgery (within 3 months), hemorrhage, anemia, hypomagnesemia, zinc deficiency, organ transplantation (lung or cardiac), plasma exchange, or concurrent use of medications known to lower ALP (corticosteroids, chemotherapy, or antiresorptive agents). Elimination criteria: patients with incomplete clinical or biochemical data that precluded adequate phenotypic evaluation were additionally eliminated from the final analysis.

Data collection included demographic variables (age and sex), detailed medical history including fracture history, fatigue, recurrent MSK pain, anxiety, depression, dental abnormalities, renal stones, degenerative joint disease, past medical history, medication history (particularly antiresorptive agents), family history of HPP, biochemical parameters, DXA results, levels of TNSALP substrates, and results of genetic testing, if available. MSK symptoms were systematically recorded and included: chronic MSK pain (encompassing back pain, joint pain, and limb pain), muscle weakness, arthralgia, reduced mobility or functional limitation, and chondrocalcinosis documented on imaging.

Genetic testing and measurement of TNSALP substrates, such as pyridoxal-5′-phosphate (PLP), were not routinely available and therefore were not systematically performed. Importantly, this study does not claim a confirmed diagnosis of HPP in any patient. Rather, patients were classified as having “unexplained persistently low ALP potentially compatible with HPP” based on three criteria: [1] two or more ALP measurements below the lower reference limit separated by ≥ 30 days [2], absence of identifiable secondary causes after comprehensive clinical and laboratory review, and [3] the presence of one or more clinical features recognized in adult HPP, in accordance with the diagnostic framework proposed by Khan et al. (2024). Formal confirmation of HPP would require measurement of PLP and/or *ALPL* genetic testing, which were not available in this retrospective setting.

Ethical approval for this study was obtained from the Institutional Review Board of King Abdullah International Medical Research Center (KAIMRC), Riyadh, Saudi Arabia. The study was conducted in accordance with the ethical principles of the Declaration of Helsinki. This study received no funding from the hospital or external sources.

### Statistical analysis

Data were entered into a secure database and analyzed using IBM SPSS Statistics for Windows, Version 28.0 (IBM Corp., Armonk, NY, USA). Continuous variables were assessed for normality using the Shapiro–Wilk test. Normally distributed variables were expressed as means ± standard deviations (SD), and non-normally distributed variables as medians with interquartile ranges (IQR). Categorical variables were summarized as frequencies and percentages.

Correlations between ALP levels (lowest, highest, and mean) and bone mineral density (BMD) parameters from DXA scans were evaluated using Pearson correlation coefficients for normally distributed data and Spearman rank correlation coefficients for non-normally distributed data.

Given the small number of patients with persistently low ALP, analyses were primarily descriptive. Comparisons between subgroups were performed using appropriate univariate tests. Statistical significance was set at *p* < 0.05.

## Results

**Fig. 1:**
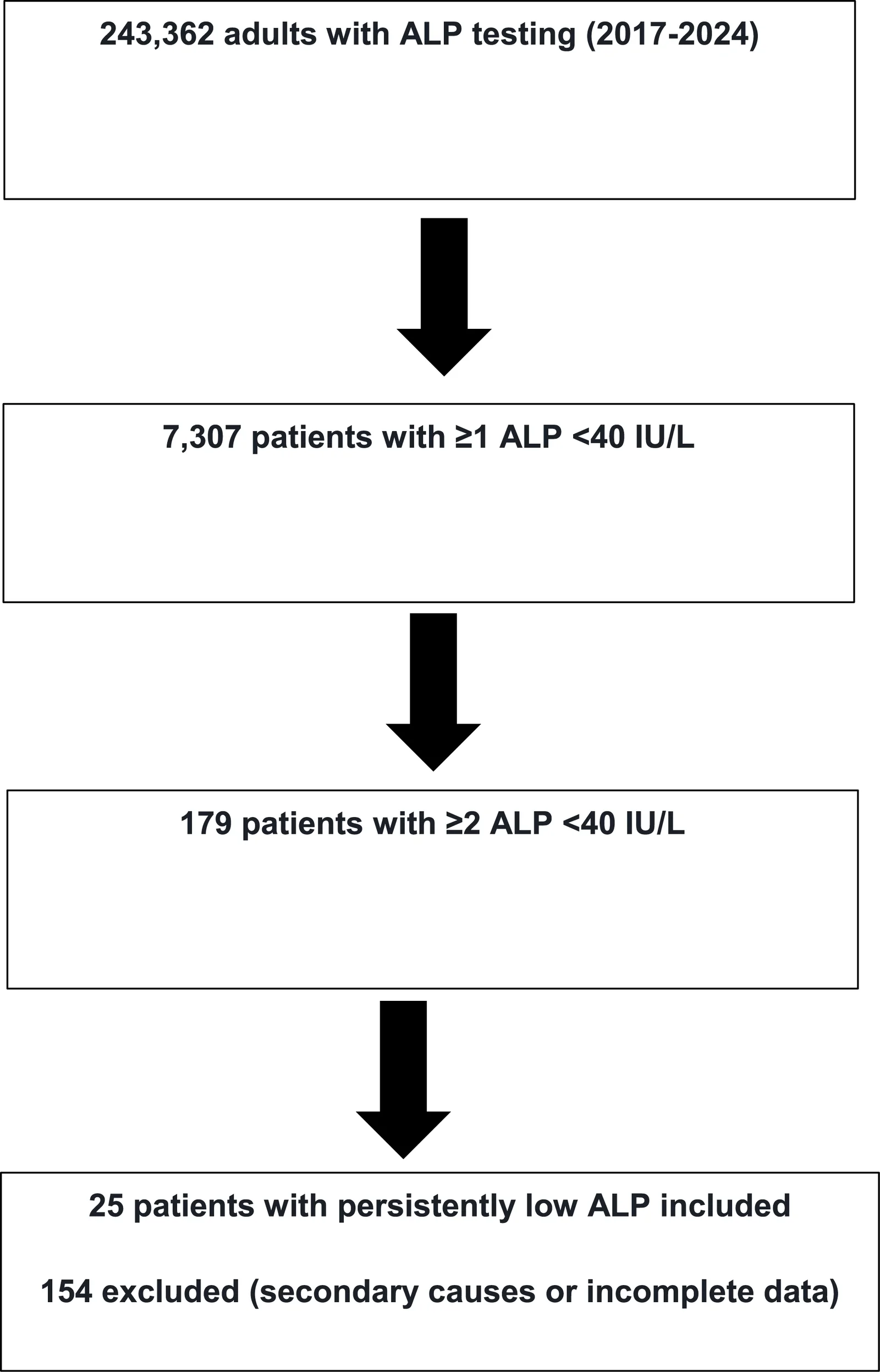
Study flowchart showing patient selection and exclusion criteria

Over the seven-year study period (2017–2024), a total of 928,403 alkaline phosphatase (ALP) tests were performed for 243,362 patients. Of these, 7,307 patients (3.0%) had at least one ALP measurement below the lower limit of reference range (40 IU/L). Within this subgroup, 179 patients had ≥ 2 low ALP values. Following a comprehensive evaluation, 154 patients were excluded because of incomplete clinical or biochemical data or the presence of identifiable secondary causes of low ALP. This resulted in the identification of 25 patients who met the study definition of persistently low ALP (Fig. 1). Among the 154 excluded patients, the most common reasons for exclusion were incomplete clinical or biochemical data (*n* = 76, 49.4%), followed by hypothyroidism (*n* = 46, 29.9%), zinc deficiency (*n* = 16, 10.4%), antiresorptive therapy including bisphosphonates or denosumab (*n* = 9, 5.8%), and corticosteroid use (*n* = 7, 4.5%) (Table 1). These findings are consistent with recognized secondary causes of reduced ALP activity and highlight the importance of excluding potentially reversible or treatment-related etiologies before considering hypophosphatasia as a diagnostic possibility.

**Table 1:** Causes of exclusion among patients with repeated low ALP values (*n* = 154)

Cause of Exclusion	*n* (%)
Incomplete clinical/biochemical data	76 (49.4)
Hypothyroidism	46 (29.9)
Zinc deficiency	16 (10.4)
Antiresorptive therapy	9 (5.8)
Corticosteroid use	7 (4.5)
Total	154 (100)

The mean age of the study cohort was 52.2 years (range: 18–92 years), with a predominance of female patients (68%, *n* = 17). All patients met the study definition of persistently low ALP based on age- and sex-specific reference ranges. Twenty-four patients (96%) had a nadir ALP < 40 IU/L, while one younger patient met the definition based on age-specific reference ranges. In the present cohort, the mean of the lowest ALP values was 30.2 IU/L (range: 18–41 IU/L), while the mean ALP across all available measurements per patient was 34.9 IU/L.

Biochemical evaluation showed a mean corrected calcium level of 2.27 mmol/L (SD ± 0.08), a mean phosphate level of 1.22 mmol/L (SD ± 0.14), a mean vitamin D level of 59.6 nmol/L (range: 21–115 nmol/L), and a mean magnesium level of 0.78 mmol/L (SD ± 0.09) (Table 2).

**Table 2:** Baseline demographic, biochemical, and clinical characteristics of the study cohort

Characteristic	Mean ± SD or *n* (%)	Range
Age (years)	52.2	18–92
Female sex, n (%)	17 (68%)	—
Lowest ALP (IU/L)	30.2	18–41
Mean ALP (IU/L)	34.9	—
Corrected calcium (mmol/L)	2.27	± 0.08
Phosphate (mmol/L)	1.22	± 0.14
Vitamin D (nmol/L)	59.6	21–115
Magnesium (mmol/L)	0.78	± 0.09
Musculoskeletal (MSK) symptoms, n (%)	22 (92%)	—
Fatigue, n (%)	19 (79%)	—
Dental abnormalities, n (%)	9 (50%)	—
Anxiety, n (%)	7 (33%)	—
Depression, n (%)	6 (29%)	—
Renal stones, n (%)	1 (4%)	—

Musculoskeletal (MSK) symptoms were the most prevalent clinical manifestation. MSK symptom data were available for 24 patients, of whom 22 (92%) were affected. These manifestations included chronic musculoskeletal pain (back pain, joint pain, and limb pain), muscle weakness, arthralgia, reduced mobility or functional limitation, and radiographic evidence of chondrocalcinosis. Fatigue data were available for 24 of 25 patients, of whom 19 (79%) were affected. Dental abnormality data were available for 18 of 25 patients (those with accessible dental records), of whom 9 (50%) were affected. Anxiety and depression data were available for 21 of 25 patients (those with documented psychiatric history), of whom 7 (33%) and 6 (29%) were affected, respectively. Nephrolithiasis was rare, documented in only 4% (1/22) of patients with available urological history. Subgroup analysis demonstrated that patients with MSK symptoms had significantly lower ALP values compared with those without. The mean lowest ALP level was 29.4 ± 5.4 IU/L in patients with MSK manifestations versus 39.5 ± 2.1 IU/L in those without (*p* = 0.021). Correlation analysis demonstrated a moderate inverse association between lowest ALP and MSK symptoms (*r* = − 0.49, *p* = 0.016). In addition, fatigue was also associated with significantly lower nadir ALP levels (*p* = 0.019), whereas dental abnormalities and depression did not show significant correlations with ALP levels. (Table 2).

DXA data were available for 10 patients. The mean lumbar spine T-score was − 0.93 (range − 3.6 to + 3.1). Half of the patients had T-scores ≤ − 1.6, consistent with osteopenia or osteoporosis. The mean femoral neck T-score was − 1.23 (range: −2.6 to + 0.2). Correlation analysis demonstrated a weak positive association between lowest ALP and vitamin D levels, as well as femoral neck T-scores. A weaker relationship was also observed with lumbar spine T-scores and corrected calcium. Vitamin D levels showed mild positive correlations with both mean ALP and bone mineral density parameters, while phosphate concentrations were not meaningfully correlated with DXA outcomes.

Fracture history was documented in 11 of 24 patients (46%) in whom fracture data were available (one patient had no fracture history recorded in the electronic medical record), most commonly involving the vertebrae, hip, and metatarsals. Several patients sustained multiple fractures, with reports of poor healing or delayed union, including cases necessitating surgical intervention such as hip replacement. The mean lowest ALP level among patients with a fracture was 29.1 ± 5.5 IU/L, compared with 31.2 ± 5.0 IU/L in those without fractures; however, this difference was not statistically significant (*p* = 0.56). Correlation analysis likewise revealed a weak negative association between lowest ALP and fracture occurrence (*r* = − 0.13, *p* = 0.56).

In addition, most patients had undergone several imaging studies, including plain radiographs, CT, and MRI, often in the context of persistent MSK pain or functional symptoms. Despite this, radiology reports were generally unremarkable or nonspecific, and none documented recognition of low ALP or suspicion for HPP. Importantly, persistently low ALP values, a potential biochemical hallmark of HPP, were consistently overlooked and not explicitly mentioned in patients’ electronic medical records, underscoring a diagnostic gap in clinical recognition.

Once a patient with persistently low ALP was identified, we extended our review to include available clinical and biochemical data from their family members. This assessment revealed that several relatives of index patients also demonstrated persistently low ALP values. Although detailed clinical information was not consistently available for all relatives, in our cohort, 6 index patients (25%) had family members with documented persistently low ALP values.

## Discussion

The findings of this large retrospective study are consistent with previous reports that persistently low ALP is often overlooked in routine hospital-based practice [7, 8]. In our cohort, 3% had at least one ALP value < 40 IU/L, and the prevalence of persistently low ALP levels (defined as having two or more ALP values < 40 IU/L) was 0.01%. This prevalence is comparable to previous estimates from French [8] and Brazilian cohorts [9] but lower than the large rural U.S. cohort using a stricter threshold of < 30 IU/L. While this discrepancy may reflect different thresholds and selection criteria, it also highlights a critical diagnostic gap in the recognition of persistently low ALP.

Among adults with persistent low ALP in the absence of secondary causes, a substantial burden of symptoms potentially consistent with HPP was observed: MSK symptoms and fatigue were the most frequent presentations, affecting more than 90% and 79% of the cohort, respectively, and both were associated with lower ALP levels. Half of the patients had dental abnormalities, and approximately one-third had depression. These extra-skeletal manifestations mirror patterns described in confirmed HPP patients, reinforcing their diagnostic relevance. Colazo et al. (2019) reported fatigue, dental problems, and depression in 66%, 77%, 39%, respectively, of confirmed HPP patients [10]. Similarly, Seefried et al. (2020), analyzing data from the Global HPP Registry, demonstrated that these symptoms are increasingly recognized as key findings in adult patients with HPP, highlighting its importance as a core symptom of HPP rather than a nonspecific complaint [11].

Almost half of the patients reported fracture history, frequently involving spine, hips, or metatarsals. Although the difference in ALP levels between fracture and non-fracture groups did not reach statistical significance, this aligns with the clinical heterogeneity of HPP, where fracture risk is not always predicted by ALP values alone [12]. In our subset with BMD testing, a weak positive association between low ALP and osteopenia/osteoporosis, particularly at the femoral neck, was observed. Genest et al. (2021) reported that lumbar spine BMD was associated with an increased risk of HPP-related fractures, whereas hip BMD remained a more reliable predictor of fracture risk [12]. Thus, DXA alone may be insufficient to characterize skeletal fragility in this population.

In our cohort, one-quarter of patients had relatives with persistently low ALP values, suggesting possible familial clustering. Although detailed clinical data for these relatives were limited, this observation is consistent with the hereditary nature of HPP; perinatal and infantile forms are typically inherited in an autosomal recessive pattern, while milder adult and odontohypophosphatasia can follow autosomal dominant or recessive inheritance, depending on the pathogenic effect of the *ALPL* variants [13, 14].

Family mapping studies with biochemical and genetic testing have been shown to improve diagnostic yield and identify subclinical disease. For instance, Beck et al. (2022) demonstrated that heterozygous *ALPL* variants identified through expanded carrier screening were frequently associated with clinical or biochemical features of HPP [15]. Similarly, Kannu et al. (2025) highlighted multigenerational pedigrees in six families, illustrating how biochemical and genetic screening across relatives can uncover subclinical or asymptomatic carriers [16], further supporting the role of familial screening in recognizing underdiagnosed disease, allowing early diagnosis and avoidance of inappropriate therapies.

It is important to emphasize that our study reflects a biochemical signal rather than definitive HPP. As in prior large series [7, 8], confirmatory tests such as serum PLP or *ALPL* gene sequencing were not performed; therefore, the true prevalence of HPP in this population remains unknown. Nevertheless, in cohorts where genetic testing has been pursued, a substantial proportion of adults with persistent low ALP values were found to harbor pathogenic *ALPL* variants: 50% in patients with unexplained low ALP [17], 57% in a rheumatology cohort [18], and 67% rising to 80% when a structured diagnostic algorithm was applied in a mixed adult–pediatric cohort [19]. These observations emphasize that, in the absence of secondary causes, more than half of genetically tested patients may have HPP. Therefore, persistently low ALP should prompt further evaluation, including measurement of PLP and consideration of *ALPL* genetic testing when clinically appropriate.

Our study has notable strengths, including its large sample size, real-world relevance, and the use of repeated ALP measurements over a long follow-up period, which enabled the identification of actual persistent low ALP cases. However, this study has several limitations. First, its retrospective design relied on electronic medical records, which may have resulted in incomplete clinical documentation. Second, confirmatory testing for hypophosphatasia, including measurement of PLP or genetic analysis of the *ALPL* gene, was not systematically performed. Therefore, the presence of HPP in this cohort cannot be definitively established. Third, bone mineral density data were available only in a subset of patients, limiting the ability to draw conclusions regarding skeletal fragility.

## Conclusion

This large hospital-based study demonstrates that persistently low ALP is rare but clinically significant. Many patients exhibited clinical features overlapping with those described in adult hypophosphatasia. The finding of familial clustering and supportive evidence from recent family mapping studies highlight the genetic basis and diagnostic value of systematic family screening. Recognition of this biochemical signal is crucial for early identification of HPP, genetic counseling, and avoidance of harmful therapies such as bisphosphonates. Future studies integrating biochemical markers, genetic sequencing, and systematic family screening are warranted to refine diagnostic algorithms and estimate the true burden of HPP in adult populations.

## Data Availability

The datasets generated and/or analyzed during the current study are not publicly available due to institutional privacy regulations but are available from the corresponding author on reasonable request.
